# Supplementary material for: Features of Psychomotor Coordination in Adolescents with Neuropsychiatric Pathology Enrolled in a Standard Educational Program
Source: Brain Sci. 2022 Feb 10;12(2):245. doi: 10.3390/brainsci12020245 (PMC8870156; doi:10.3390/brainsci12020245)
Supplement: Supplementary file 1 [file brainsci-12-00245-s001.zip › brainsci-1548303-supplementary.pdf]

Table S1. Indicators of psychomotor coordination obtained during testing for the right and left hands, Me [Q1; Q3].

	Control (n = 70)				Psychiatric patients (n = 63)			
Parameters of movement	Left hand / Right hand	T	Z	p-level	Left hand / Right hand	T	Z	p-level
Movement cycle duration	1.08 [0.97;1.14]	779	2.712	0.007	0.73 [0.50;0.96]	850	0.887	0.375
	1.12 [0.96;1.06]				0.70 [0.50;0.96]			
Time to change the motor stereotype	3.59 [2.37;4.79]	847.5	2.312	0.021	1.93 [1.35; 3.05]	622	2.485	0.013
	3.70 [2.70;5.89]				2.48 [1.68;4.90]			
Time of the reaction to visual stimulus	0.16 [0.14;0.19]	884	0.649	0.517	0.18 [0.16;0.21]	882	0.456	0.648
	0.16 [0.14;0.18]				0.17 [0.16;0.22]			
Time of the reaction to acoustic stimulus	0.16 [0.14;0.19]	977	0.821	0.412	0.18 [0.15;0.23]	784.5	0.550	0.582
	0.17 [0.14;0.19]				0.18 [0.15;0.23]			
Error of correction of flexors	1.68 [1.34;2.56]	1067	0.840	0.401	4.07 [3.17;5.30]	548	3.004	0.003
	1.82 [1.15;3.51]				2.76 [1.58;4.27]			
Error of correction of extensors	2.13 [1.43; 3.59]	793	2.478	0.013	2.95 [1.83;4.84]	764	1.490	0.136
	1.78 [1.21;2.54]				3.88 [2.79;4.85]			
Smoothness of movement	92.03 [88.65;94.27]	1021	1.296	0.195	87.21 [76.48;90.08]	776	1.406	0.160
	91.54 [87.34;94.23]				86.02 [69.5;89.66]			

Note to Table S1: Using Wilcoxon Matched Pairs Test.

Table S2. Comparison of measures of psychomotor coordination and sensorimotor reactivity in "Psychiatric patients" and "Control" in early and late adolescence (Mann–Whitney U test).

Parameters of movement	Early adolescence					Late adolescence				
	Control /n =22<br>Psychiatric patients / n=17	U	Z	p-level	d_{Kohen}	Control /n=48<br>Psychiatric patients /n= 46	U	Z	p-level	d_{Kohen}
Movement cycle duration	1.16 [1.12; 4.36]<br>0.98 [0.80; 2.52]	46	3.993	<0.0001	1.663	1.09 [1.04; 3.74]<br>00.98 [0.71; 2.89]	678	3.222	0.001	0.705
Time to change the motor stereotype	5.54 [4.45; 0.16]<br>3.40 [2.41; 0.19]	66	3.427	0.0003	1.313	5.01 [3.73; 0.16]<br>3.52 [2.62; 0.20]	692	3.116	0.002	0.679
Time of the reaction to visual stimulus	0.16 [0.15; 0.18]<br>0.18 [0.17; 0.21]	98	-2.520	0.011	0.882	0.16 [0.15; 0.18]<br>0.17 [0.16; 0.21]	735	-2.659	0.008	0.605
Time of the reaction to acoustic stimulus	0.18 [0.16; 0.19]<br>0.19 [0.18; 0.20]	132	-1.543	0.124	0.515	0.16 [0.13; 0.18]<br>0.17 [0.16; 0.21]	769	-2.530	0.011	0.542
Error of correction of flexors	1.69 [1.26; 2.24]<br>3.77 [2.92; 5.09]	32	-4.390	<0.0001	1.977	1.91 [1.47; 3.55]<br>3.37 [2.624.45]	605	-3.774	<0.0001	0.845
Error of correction of extensors	1.51 [1.21; 2.22]<br>3.74 [3.04; 5.61]	41	-4.135	<0.0001	1.767	2.24 [1.70; 3.51]<br>3.27 [2.58; 4.38]	675	-3.245	0.001	0.730
Smoothness of movement	91.89 [89.54; 94.22]<br>87.03 [81.68; 90.35]	60	3.597	0.0002	1.409	91.99 [87.84; 93.51]<br>83.07 [66.58; 89.50]	531	4.334	<0.0001	0.999
TRV/TRA	1.07 [0.98; 1.21]<br>1.05 [0.92; 1.13]	150	1.048	0.305	0.340	0.98 [0.89; 1.10]<br>1.01 [0.90; 1.12]	1023	-0.446	0.660	0.063
Asymmetry	7.38 [-3.65; 25.06]<br>13.66 [10.26; 23.45]	138	-1.388	0.171	0.456	3.13 [-16.51;20.85]<br>-0.26 [-9.80; 9.77]	1017	-0.143	0.890	0.136

Note to Table S2: The blue cells show Me [Q1; Q3} for the "Control".

Table S3. Comparison of measures of psychomotor coordination and sensorimotor reactivity in adolescents of early and late age in "Control" and in "Psychiatric patients" (Mann–Whitney U test)

	Control					Psychiatric patients				
Parameters of movement	Early /n = 22<br>Late /n = 48	U	Z	p-level	d_{Kohen}	Early / n = 17<br>Late / n = 46	U	Z	p-level	d_{Kohen}
Movement cycle duration	1.16 [1.12; 4.36]<br>1.09 [1.04; 3.74]	206	4.074	<0.0001	1.115	0.98 [0.80; 2.52]<br>0.98 [0.71; 2.89]	358	0.511	0.618	0.129
Time to change the motor stereotype	5.54 [4.45; 0.16]<br>5.01 [3.73; 0.16]	394.5	1.689	0.091	0.414	3.40 [2.41; 0.19]<br>3.52 [2.62; 0.20]	341.5	-0.766	0.447	0.196
Time of the reaction to visual stimulus	0.16 [0.15; 0.18]<br>0.16 [0.15; 0.18]	492	-0.322	0.754	0.109	0.18 [0.17; 0.21]<br>0.17 [0.16; 0.21]	363.5	0.426	0.673	0.107
Time of the reaction to acoustic stimulus	0.18 [0.16; 0.19]<br>0.16 [0.13; 0.18]	382	1.847	0.065	0.453	0.19 [0.18; 0.20]<br>0.17 [0.16; 0.21]	331	0.929	0.360	0.236
Error of correction of flexors	1.69 [1.26; 2.24]<br>1.91 [1.47; 3.55]	417	-1.404	0.163	0.341	3.77 [2.92; 5.09]<br>3.37 [2.624.45]	317	1.146	0.258	0.292
Error of correction of extensors	1.51 [1.21; 2.22]<br>2.24 [1.70; 3.51]	307	-2.796	0.005	0.709	3.74 [3.04; 5.61]<br>3.27 [2.58; 4.38]	315	1.177	0.245	0.300
Smoothness of movement	91.89 [89.54; 94.22]<br>91.99 [87.84; 93.51	456	0.911	0.368	0.219	87.03 [81.68; 90.35]<br>83.07 [66.58; 89.50]	335	0.867	0.394	0.220
TRV/TRA	1.07 [0.98; 1.21]<br>0.98 [0.89; 1.10]	344.5	2.221	0.026	0.579	1.05 [0.92; 1.13]<br>1.01 [0.90; 1.12]	348	0.666	0.514	0.168
Asymmetry	7.38 [-3.65; 25.06]<br>3.13 [-16.51;20.85]	393	1.481	0.141	0.417	13.66 [10.26; 23.45]<br>-0.26 [-9.80; 9.77]	205	2.801	0.004	0.779

Note to Table S3: The blue cells show Me [Q1; Q3] for the early adolescents

Table S4. Comparison of measures of psychomotor coordination and sensorimotor reactivity in “Control”, “Organic disorders patients” and “Without Organic disorders patients” (Mann–Whitney U test)

	Groups			Comparison groups											
Parameters of movements	C n=70	O n=36	WO n=27	C *vs* O				C *vs* WO				O *vs* WO			
	Me [Q1; Q3]			U	Z	p	d	U	Z	p	d	U	Z	p	d
Movement cycle duration	1.07 [0.96; 1.12]	0.72 [0.49; 0.92]	0.72 [0.49; 1.20]	513	4.98	< 0.0001	1.14	648	2.39	0.016	0.43	449	-0.51	0.615	0.19
Time to change the motor stereotype	4.02 [2.79; 5.12]	2.45 [1.77; 3.76]	2.64 [1.82; 3.23]	673	3.91	< 0.0001	0.88	515	3.46	< 0.0001	0.67	481	0.07	0.951	0.12
Time of the reaction to visual stimulus	0.16 [0.15; 0.18]	0.17 [0.15; 0.19]	0.21 [0.17; 0.24]	966	-1.86	0.063	0.33	411	-4.24	< 0.0001	1.11	287	-2.77	0.005	0.96
Time of the reaction to acoustic stimulus	0.17 [0.15; 0.19]	0.17 [0.15; 0.19]	0.19 [0.17; 0.29]	1094	-1.11	0.271	0.21	511	-3.49	< 0.0001	0.82	324	-2.25	0.024	0.68
Error of correction of flexors	1.74 [1.46; 2.99]	3.31 [2.55; 4.36]	3.56 [2.92; 5.25]	576	-4.56	< 0.0001	0.96	383	-4.52	< 0.0001	1.07	384	-1.42	0.160	0.42
Error of correction of extensors	1.96 [1.51; 2.98]	3.13 [2.51; 4.21]	4.02 [2.92; 5.61]	647	-4.09	< 0.0001	0.87	418	-4.24	< 0.0001	0.95	388	-1.36	0.177	0.35
Smoothnes s of movement	91.99 [88.33; 93.71]	85.14 [70.29; 89.35]	85.58 [59.25; 90.99]	512	4.99	< 0.0001	1.13	453	3.96	< 0.0001	0.84	462	-0.33	0.746	0.15
TRV/TRA	1.01 [0.94; 1.13]	1.03 [0.90; 1.12]	1.02 [0.88; 1.22]	1212	0.21	0.838	0.03	914	0.14	0.89	0.096	481	0.069	0.951	0.12
Asymmetr y	-0.44 [-11.53; 21.21]	2.28 [-7.18; 13.52]	4.41 [-5.13; 19.02]	1216	-0.05	0.959	0.07	817	-0.56	0.576	0.104	432	-0.514	0.615	0.17

Note to Table S4: Groups: Control (C), Organic disorders patients (O), Without Organic disorders patients (WO). d – Kohen coefficient for Mann–Whitney U test.
